# Effects of Weight Loss Speed on Kidney Function Differ Depending on Body Mass Index in Nondiabetic Healthy People: A Prospective Cohort

**DOI:** 10.1371/journal.pone.0143434

**Published:** 2015-11-23

**Authors:** Eiichiro Kanda, Toshitaka Muneyuki, Kaname Suwa, Kei Nakajima

**Affiliations:** 1 Department of Nephrology, Tokyo Kyosai Hospital, Meguro, Tokyo, Japan; 2 Center for life science and bioethics, Tokyo Medical and Dental University, Bunkyo, Tokyo, Japan; 3 Department of Rehabilitation, Funabashi City Rehabilitation Hospital, Funabashi, Chiba, Japan; 4 Saitama Health Promotion Corporation, Hikigun, Saitama, Japan; 5 Department of Metabolism, Kuki General Hospital, Kuki, Saitama, Japan; University of Catanzaro Magna Graecia, ITALY

## Abstract

**Background:**

Obesity is associated with diabetes mellitus and cardiovascular diseases. However, it has been reported that weight loss is associated with incident chronic kidney disease (CKD) in healthy males. The purpose of this prospective cohort study is to investigate the effects of weight loss on kidney function in healthy people in terms of body mass index (BMI) and gender.

**Methods:**

A total of 8447 nondiabetic healthy people were enrolled in the Saitama Cardiometabolic Disease and Organ Impairment Study, Japan. Relationships between estimated glomerular filtration rate (eGFR) change, BMI, and BMI change were evaluated using 3D-scatter plots with spline and generalized additive models (GAMs) adjusted for baseline characteristics.

**Results:**

The subjects were stratified into four groups according to BMI. The mean±standard deviations for males and females were, respectively, 40.11±9.49, and 40.3±9.71 years for age and 76.39±17.72 and 71.49±18.4 ml/min/1.73m^2^ for eGFR. GAMs showed that a decreasing BMI change (<-1 kg/m^2^/year) was associated with a decreasing eGFR change in males with high normal BMIs (22 kg/m^2^≤BMI<25 kg/m^2^). A decreasing BMI change (<-2 kg/m^2^/year) was associated with an increasing eGFR change in overweight males (25 kg/m^2^≤BMI). Among underweight females (BMI<18.5 kg/m^2^), decreasing BMI was observed with decreasing eGFR.

**Conclusions:**

These findings suggest that the benefit and risk of weight loss in relation to kidney function differs depending on BMI and weight loss speed, especially in males.

## Introduction

Obesity is associated with progression of chronic kidney disease (CKD), and impairs kidney function, namely, obesity-related glomerulopathy (ORG). Focal segmental glomerulosclerosis and glomerulomegaly are observed among obese people [1–3]. The Framingham heart study and a prospective cohort study in Japan showed that obesity is associated with the increase in the risk of incident CKD [4, 5]. The Atherosclerosis Risk in Communities (ARIC) Study showed that metabolic syndrome is associated with incident CKD in nondiabetic adults [6]. A systematic review showed that obesity are associated with incident CKD [7].

Weight loss can improve many of the obesity-related risk factors for cardiovascular disease [8]. However, weight loss is not always beneficial for kidney function. U-shaped associations of body mass index (BMI) with incident proteinuria and microalbuminuria have been reported [9, 10]. A cohort study from Korea also showed a u-shaped association between weight change and incident CKD [11]. Another cohort study in Japan showed that percent change in BMI (< 1%) is associated with incident CKD [12]. These studies suggest that weight loss may impair kidney function.

Obese people need to lose weight to prevent diabetes mellitus (DM) and cardiovascular diseases (CVDs). However, as described above, whether weight loss is good for their kidney function or not has not been established yet. To provide guidance on a safe weight loss to obese people, more lines of evidence of the relationship between weight loss and kidney function are needed. Moreover, the previous studies mainly were based on the data of males [11, 12]. Because there are differences in muscle mass and lifestyle between genders, the content of the guidance is determined by gender and obesity.

The Saitama Cardiometabolic Disease and Organ Impairment Study (SCDOIS) in Japan is a community-based prospective cohort study [9, 13]. We examined nonlinearly the effects of BMI change and loss of kidney function considering BMI and gender using the SCDOIS data.

## Materials and Methods

### Data Source

SCDOIS was a multidisciplinary observational epidemiological research study conducted in Saitama prefecture, Japan [9, 13]. In brief, this study started in 2011 and involved three institutions in Saitama, namely, Josai University, Jichi University, and Saitama Health Promotion Corporation. The protocol of this study was in accordance with the Declaration of Helsinki, and was approved by the ethics committees of Josai University, Jichi University, and Saitama Health Promotion Corporation. Written informed consent regarding participation to this study was obtained at the time of the checkup from all the subjects. Through this study, community-based data from medical checkups of asymptomatic people have been obtained.

In this study, we analyzed data followed up for three years collected from records of the medical checkups of asymptomatic people living or working in Saitama from 1999 to 2008. The study population consisted of 104796 subjects. Individuals with missing data such as age, gender, and serum creatinine level were excluded from this study. And the patients with DM, infection and malignancies were also excluded. Among 29782 subjects, the 8447 subjects followed up for three years were included in this study. Finally, the analysis dataset was composed of the data based on two points, baseline and 3-years later.

Baseline patient data, including age; gender; BMI; serum creatinine levels; proteinuria; comorbid conditions of hypertension, and dyslipidemia; histories of CVDs; and the habit of smoking were collected from all the subjects. Data on age, BMI, and serum creatinine level were collected at baseline and three years later. eGFR was calculated using the following equation for the Japanese population of the Japanese Society of Nephrology [14]: eGFR (ml/min/1.73m^2^) = 194 × serum Cr^-1.094^ × age^-0.287^(for female) ×0.739, where Cr = serum creatinine level (mg/dl). The intervals of changes in BMI and eGFR were 3 years among all subjects. Annual GFR change was calculated using the following formula: eGFR change (ml/min/1.73m^2^/year) = (eGFR_after_ − eGFR_before_)/3. And annual BMI change was calculated using the following formula: BMI change (kg/m^2^/year) = (BMI_after_ − BMI_before_)/3. In this study, negative and positive changes in eGFR and BMI were expressed as decreasing and increasing changes, respectively. A decrease in BMI was expressed as weight loss.

### Statistical Analyses

Statistical analyses were carried out separately by gender. The subjects were classified into four groups according to their BMIs; Group 1 (underweight), BMI < 18.5 kg/m^2^; Group 2 (low normal BMI), 18.5 kg/m^2^ ≤ BMI < 22 kg/m^2^; Group 3 (high normal BMI), 22 kg/m^2^ ≤ BMI < 25 kg/m^2^; and Group 4 (overweight), 25 kg/m^2^ ≤ BMI. Data are presented as mean±standard deviation. These groups were compared using the chi-square test, and one-way analysis of variance as appropriate. The subjects were also classified into two groups according to the medians of their ages: old males, 41.8 years ≤ age; young males, age < 41.8 years; old females, 42.4 years ≤ age; and young females, age < 42.4 years. The distributions of BMI, BMI change, and eGFR change were examined using scatter plots with 3-D spline curves. The interaction between age and gender was examined using a generalized additive model (GAM) with spline including age, gender, the interaction term of age and gender, BMI, BMI change, eGFR, proteinuria, comorbid conditions of hypertension and dyslipidemia, histories of CVDs, and the habit of smoking. Moreover, by gender, we also examined the interactions between age and BMI change using GAMs with spline including BMI, BMI change, age, the interaction term of age and BMI change, eGFR, proteinuria, comorbid conditions of hypertension and dyslipidemia, histories of CVDs, and the habit of smoking. Then, by gender, in all subjects or in each BMI group, the effects of BMI and BMI change on eGFR change were examined using GAMs including BMI, BMI change, age, eGFR, proteinuria, comorbid conditions of hypertension and dyslipidemia, histories of CVDs, and the habit of smoking. Statistical analyses were performed using SAS version 9.4 (SAS Institute, Cary, NC). Values of *p* < 0.05 were considered statistically significant.

## Results

The demographics of the subjects including biochemical data are shown in Tables 1 and 2. Both males and females showed similar trends, that is, Group 4 had higher age, and larger numbers of subjects with comorbid conditions of hypertension, dyslipidemia, and histories of CVD, and CKD Stage G3 than Group 1. Group 1 showed higher eGFR and a more rapidly decreasing BMI change than Group 4. Different patterns of eGFR change were observed between males and females. In males, the decreasing eGFR change in Group 1 was more rapid than that in Group4. On the other hand, females in Groups 2 and 3 showed more rapidly decreasing eGFR changes than those in Groups 1 and 4.

**Table 1:** Baseline characteristics of gender-specific groups classified by body mass index in males.

	All	Group 1	Group 2	Group 3	Group 4	*p*
N (%)	5937	173 (2.91)	1686 (28.40)	2282 (38.44)	1796 (30.25)	
Age (years)	40.11±9.49	37.19±9.81	38.22±10.09	40.93±9.16	41.16±8.96	0.0001
BMI (kg/m^2^)	23.6±3.07	17.59±0.69	20.61±0.95	23.45±0.85	27.25±2.07	0.0001
BMI change (kg/m^2^/year)	0.09±0.37	0.11±0.23	0.14±0.32	0.1±0.35	0.04±0.43	0.0001
eGFR (ml/min/1.73m^2^)	76.39±17.72	81.72±19.75	79.93±18.84	75.15±16.99	74.05±16.68	0.0001
CKD Stage						0.0001
G1 (%)	1056 (17.79)	46 (26.59)	375 (22.24)	391 (17.13)	244 (13.58)	
G2 (%)	3652 (61.51)	100 (57.80)	1043 (61.86)	1384 (60.65	1125 (62.64)	
G3 (%)	1229 (20.70)	27 (15.61)	268 (15.90)	507 (22.22)	427 (23.78)	
eGFR change (ml/min/1.73m^2^/year)	-3.75±5.54	-4.31±6.38	-4.23±5.81	-3.58±5.35	-3.46±5.4	0.0025
Proteinuria (%)	249 (4.19)	10 (5.78)	43 (2.55)	77 (3.37)	119 (6.63)	0.0001
Hypertension (%)	321 (5.41)	7 (4.05)	46 (2.73)	114 (5.00)	154 (8.57)	0.0001
Dyslipidemia (%)	329 (5.54)	1 (0.58)	53 (3.14)	146 (6.40)	129 (7.18)	0.0001
CVD (%)	120 (2.02)	3 (1.73)	29 (1.72)	37 (1.62)	51 (2.84)	0.033
Smoking (%)	3014 (50.77)	92 (53.18)	847 (50.24)	1167 (51.14)	908 (50.56)	0.86

Male

Group 1 (underweight), BMI < 18.5 kg/m^2^; Group 2 (low normal BMI), 18.5 kg/m^2^ ≤ BMI < 22 kg/m^2^; Group 3 (high normal BMI), 22 kg/m^2^ ≤ BMI < 25 kg/m^2^; Group 4 (overweight), 25 kg/m^2^ ≤ BMI. Values are presented as mean±SD.

Abbreviations: BMI, body mass index; eGFR, estimated glomerular filtration rate; hypertension, history of hypertension; dyslipidemia, history of dyslipidemia; CVD, history of cardiovascular disease; smoking, currently smoking.

**Table 2:** Baseline characteristics of gender-specific groups classified by body mass index in females.

	All	Group 1	Group 2	Group 3	Group 4	*p*
N (%)	2510	233 (9.28)	1184 (47.17)	681 (27.13)	412 (16.41)	
Age (years)	40.3±9.71	35.29±9.28	38.67±9.59	43.17±9.16	43.38±8.88	0.0001
BMI (kg/m^2^)	22.02±3.23	17.56±0.67	20.3±0.93	23.25±0.83	27.73±2.26	0.0001
BMI change (kg/m^2^/year)	0.07±0.36	0.13±0.33	0.06±0.3	0.05±0.35	0.08±0.5	0.073
eGFR (ml/min/1.73m^2^)	71.49±18.4	74.67±17.92	72.53±18.58	70.89±19	67.51±16.47	0.0001
CKD Stage						0.0001
G1 (%)	405 (16.14)	43 (18.45)	205 (17.31)	123 (18.06)	34 (8.25)	
G2 (%)	1264 (50.36)	131 (56.22)	630 (53.21)	310 (45.52)	193 (46.85)	
G3 (%)	841 (33.50)	59 (25.33)	349 (29.48)	248 (36.42)	185 (44.90)	
eGFR change (ml/min/1.73m^2^/year)	-3.39±6.02	-2.95±6.25	-3.47±6	-3.84±6.1	-2.68±5.75	0.0017
Proteinuria (%)	76 (3.03)	9 (3.86)	19 (1.60)	21 (3.08)	27 (6.55)	0.0001
Hypertension (%)	95 (3.78)	1 (0.43)	22 (1.86)	30 (4.41)	42 (10.19)	0.0001
Dyslipidemia (%)	107 (4.26)	3 (1.29)	40 (3.38)	36 (5.29)	28 (6.80)	0.0014
CVD (%)	45 (1.79)	4 (1.72)	18 (1.52)	8 (1.17)	15 (3.64)	0.019
Smoking (%)	268 (10.68)	24 (10.30)	133 (11.23)	71 (10.43)	40 (9.71)	0.83

Female

Group 1 (underweight), BMI < 18.5 kg/m^2^; Group 2 (low normal BMI), 18.5 kg/m^2^ ≤ BMI < 22 kg/m^2^; Group 3 (high normal BMI), 22 kg/m^2^ ≤ BMI < 25 kg/m^2^; Group 4 (overweight), 25 kg/m^2^ ≤ BMI. Values are presented as mean±SD.

Abbreviations: BMI, body mass index; eGFR, estimated glomerular filtration rate; hypertension, history of hypertension; dyslipidemia, history of dyslipidemia; CVD, history of cardiovascular disease; smoking, currently smoking.

### Distributions of BMI, BMI change and eGFR change

**Fig 1:**
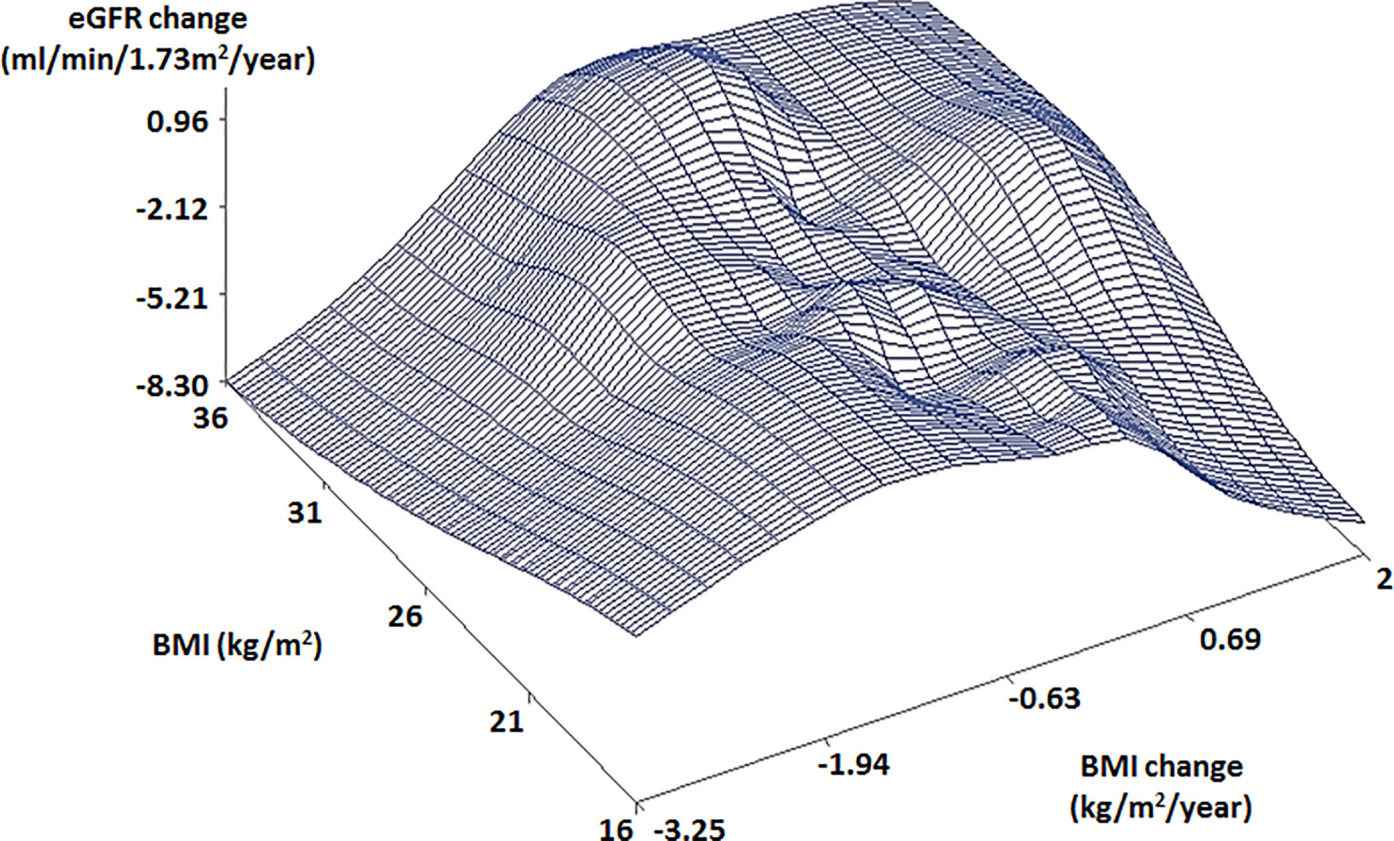
Scatter plot of relationship between BMI, BMI change, and eGFR change in old males. The subjects were 41.8 years and older. Abbreviations: BMI, body mass index; eGFR, estimated glomerular filtration rate.

**Fig 2:**
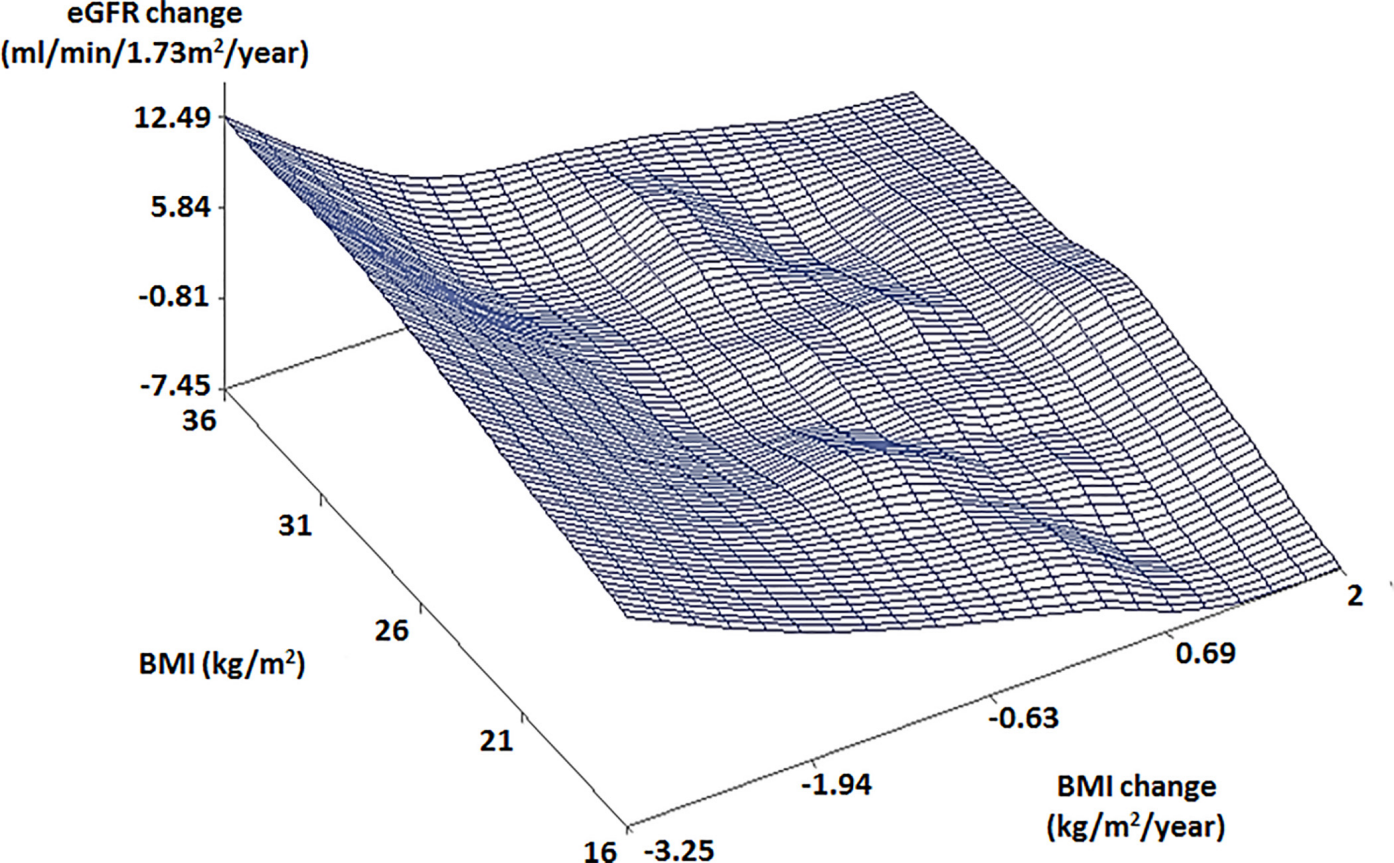
Scatter plot of relationship between BMI, BMI change, and eGFR change in young males. The subjects were younger than 41.8 years. Abbreviations: BMI, body mass index; eGFR, estimated glomerular filtration rate.

**Fig 3:**
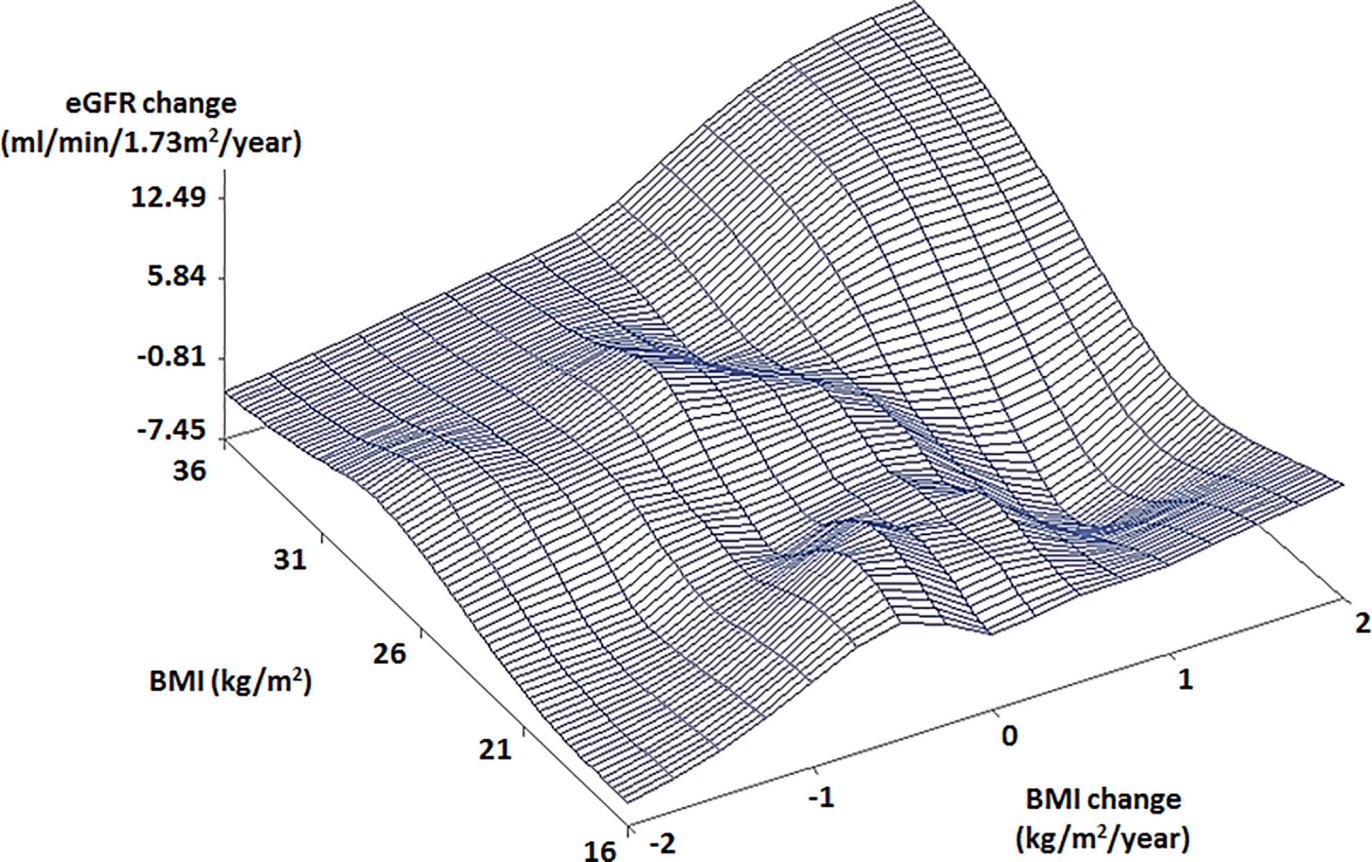
Scatter plot of relationship between BMI, BMI change, and eGFR change in old females. The subjects were 42.4 years and older. Abbreviations: BMI, body mass index; eGFR, estimated glomerular filtration rate.

**Fig 4:**
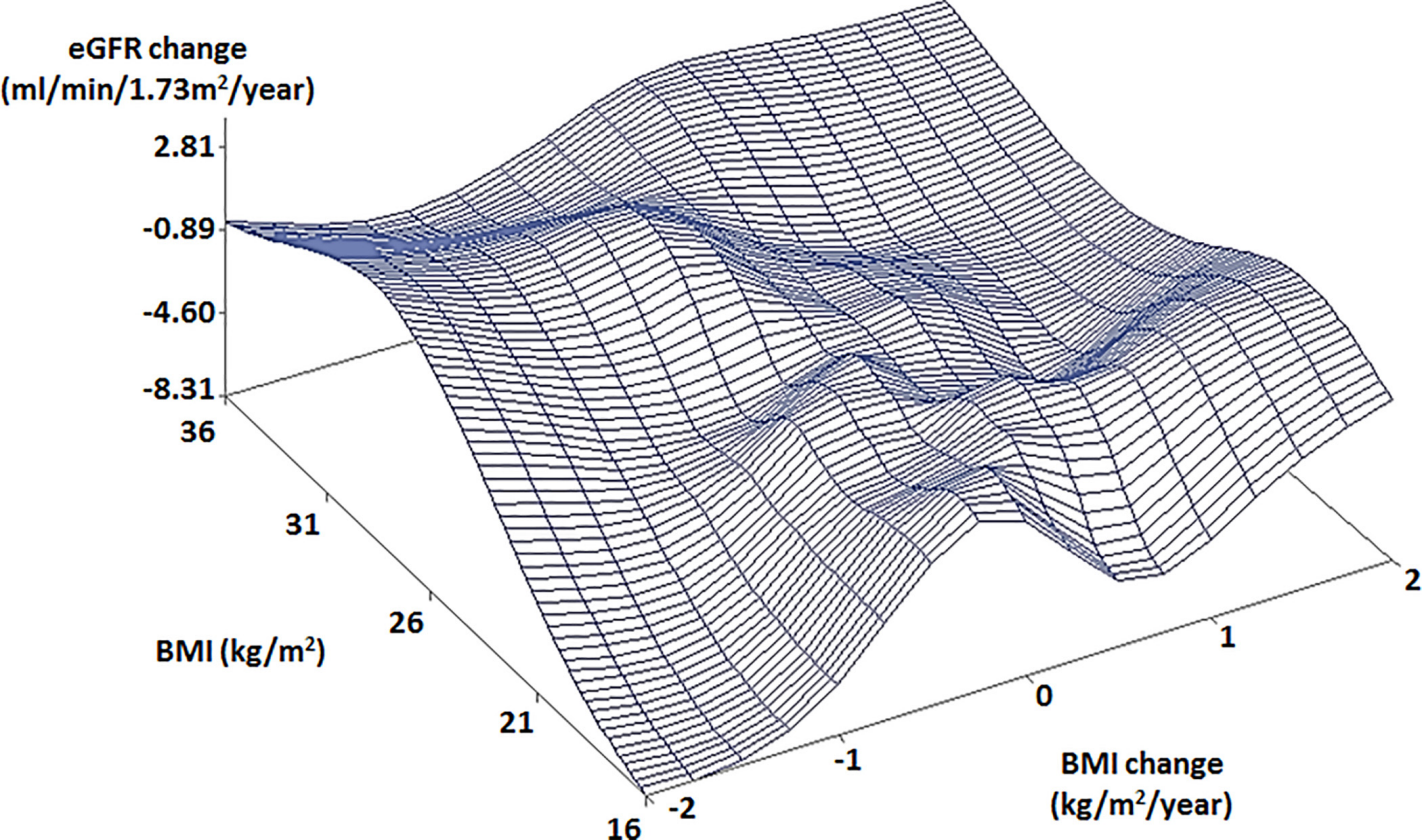
Scatter plot of relationship between BMI, BMI change, and eGFR change in young females. The subjects were younger than 42.4 years. Abbreviations: BMI, body mass index; eGFR, estimated glomerular filtration rate.

Scatter plots showed different patterns of the relationships between BMI, BMI change, and eGFR change by age and gender. In old males with high BMIs, a rapidly decreasing eGFR change was observed with a rapidly decreasing BMI change (Fig 1). In young males, an increasing eGFR change was observed with a decreasing BMI change (Fig 2). Different from males, old females with low BMI (16 kg/m^2^) showed a rapidly decreasing eGFR change with a rapidly decreasing BMI change (< -2 kg/m^2^/year) (Fig 3). Young females showed similar trends to old females (Fig 4).

### Effects of age on the relationship between eGFR change and BMI change

The interaction between age and gender was examined. A GAM including the interaction term of age and gender showed that the interaction term was not statistically significant (*p* = 0.70), and that the eGFR change was associated with the male gender (*p* = 0.0009), BMI (linear *p* = 0.020, spline *p* = 0.0011) and BMI change (linear *p* = 0.0001, spline *p* = 0.0001).

Then, the interaction between age and BMI change was also examined using GAMs. In all males, the interaction term of age and BMI change was not statistically significant (*p* = 0.059). eGFR change was associated with age (*p* = 0.0001), BMI (linear *p* = 0.021, spline *p* = 0.0001) and BMI change (linear *p* = 0.0001, spline *p* = 0.0001). In all females, eGFR was associated with age (*p* = 0.0001), and the interaction term of age and BMI change (*p* = 0.025), but not with BMI (linear *p* = 0.59, spline *p* = 0.72) and BMI change (linear *p* = 0.17, spline *p* = 0.089).

### eGFR change and BMI change

**Fig 5:**
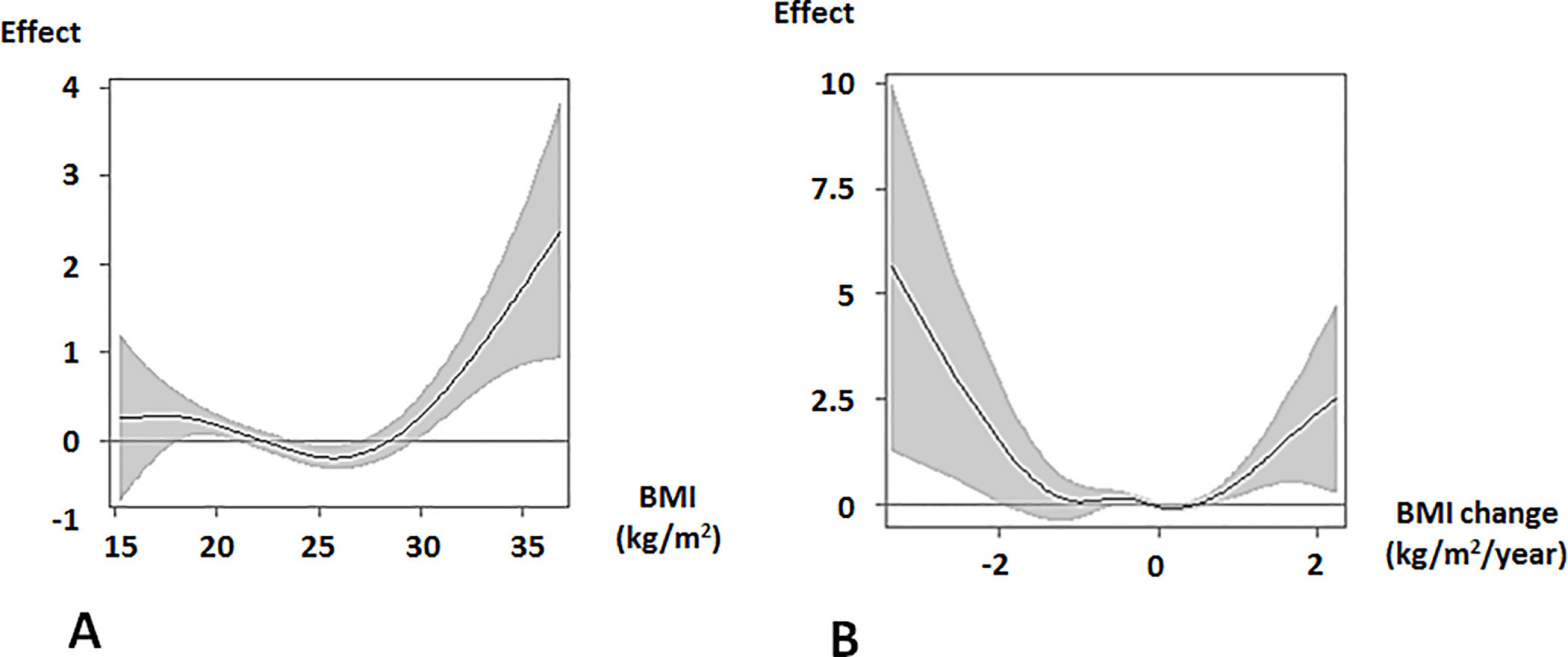
Effects of BMI and BMI change on eGFR change in males. The curve from a generalized additive model with spline shows the pattern of the effect of BMI (A) or BMI change (B) on eGFR change with 95% confidence interval. Positive and negative values of the effect indicate increasing and decreasing eGFR changes, respectively. The models were adjusted for baseline characteristics. A. Effect of BMI. An increasing eGFR change was observed for BMI < 22 kg/m^2^, and 28 kg/m^2^ ≤ BMI; linear *p* = 0.024, spline *p* = 0.0001. B. Effect of BMI change. An increasing eGFR change was observed in BMI change < -2 kg/m^2^/year, and 1 kg/m^2^/year ≤ BMI: linear *p* = 0.0001, spline *p* = 0.0001. Abbreviations: Effect, effect on eGFR change; BMI, body mass index; eGFR, estimated glomerular filtration rate.

In all males, u-shaped associations between eGFR change, BMI, and BMI change were observed. Low BMIs (< 22 kg/m^2^) and high BMIs (28 kg/m^2^ ≤) were associated with increasing eGFR changes (Fig 5A). A u-shaped association was observed between BMI change and eGFR change (Fig 5B). An increasing BMI change (1 kg/m^2^/year ≤) and a decreasing BMI change (< -2 kg/m^2^/year) were associated with increasing eGFR changes.

**Fig 6:**
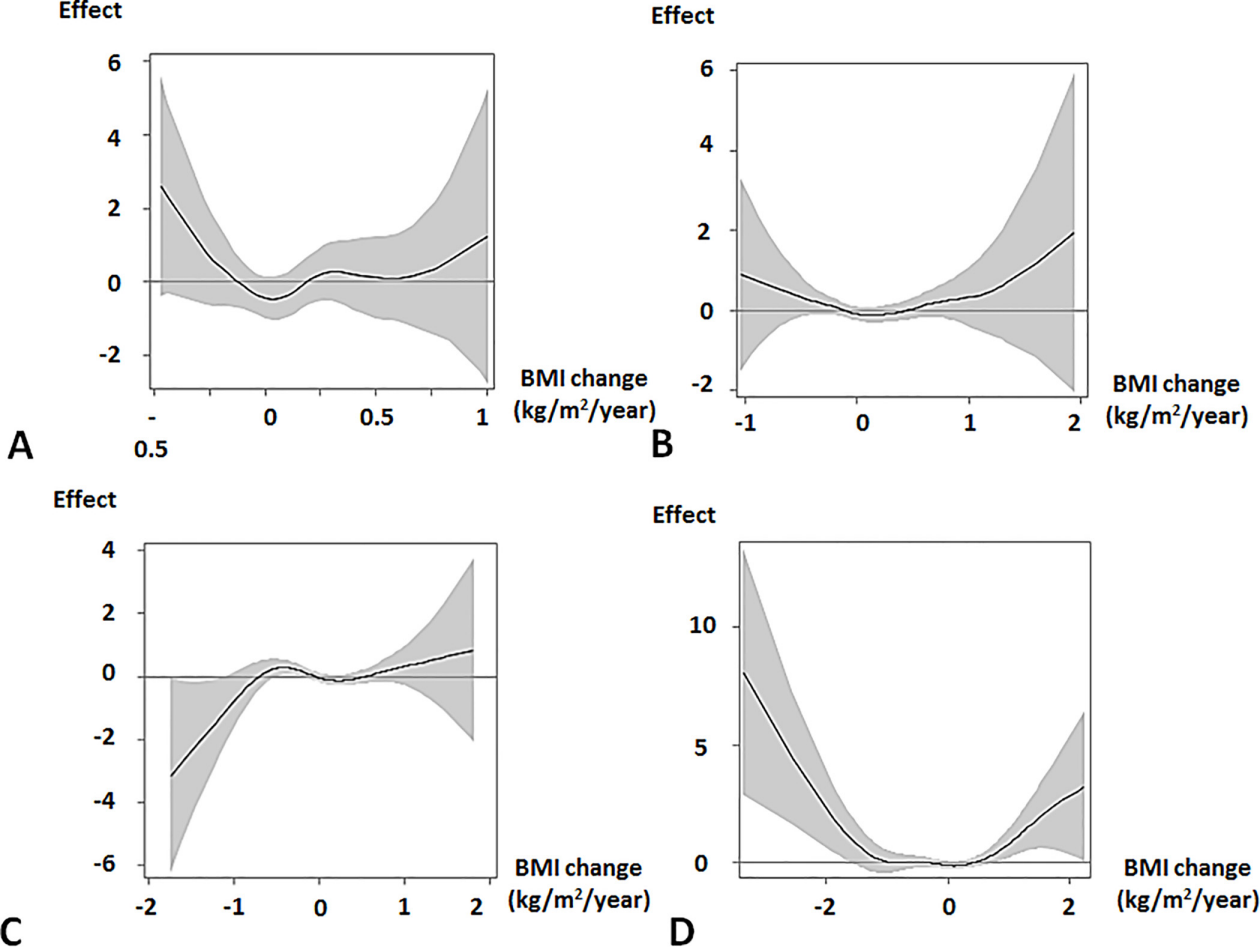
Effect of BMI change on eGFR change in males. The curve from a generalized additive model with spline shows the pattern of the effect of BMI change on eGFR change with 95% confidence interval. Positive and negative values of the effect indicate increasing and decreasing eGFR changes, respectively. The models were adjusted for baseline characteristics. Abbreviations: Effect, effect of BMI change on eGFR change; BMI, body mass index; eGFR, estimated glomerular filtration rate. A. Group 1 Linear *p* = 0.21, spline *p* = 0.18. B. Group 2 Linear *p* = 0.0001, spline *p* = 0.24. C. Group 3 Linear *p* = 0.0011, spline *p* = 0.0072. D. Group 4 Linear *p* = 0.0001, spline *p* = 0.0001.

In each BMI group of males, there was no statistically significant relationship between eGFR change and BMI. On the other hand, BMI changes were associated with eGFR changes. In Groups 1 and 2, u-shaped associations between BMI changes and eGFR changes were observed, but were not statistically significant (Fig 6A and 6B). In Group 3, a decreasing BMI change (< -1 kg/m^2^/year) was associated with a decreasing eGFR change, whereas increasing BMI (1 kg/m^2^/year ≤) was associated with an increasing eGFR change (Fig 6C). In Group 4, a u-shaped association was observed between BMI change and eGFR change (Fig 6D). An increasing BMI change (1 kg/m^2^/year ≤) and a decreasing BMI change (< -2 kg/m^2^/year) were associated with an increasing eGFR change.

**Fig 7:**
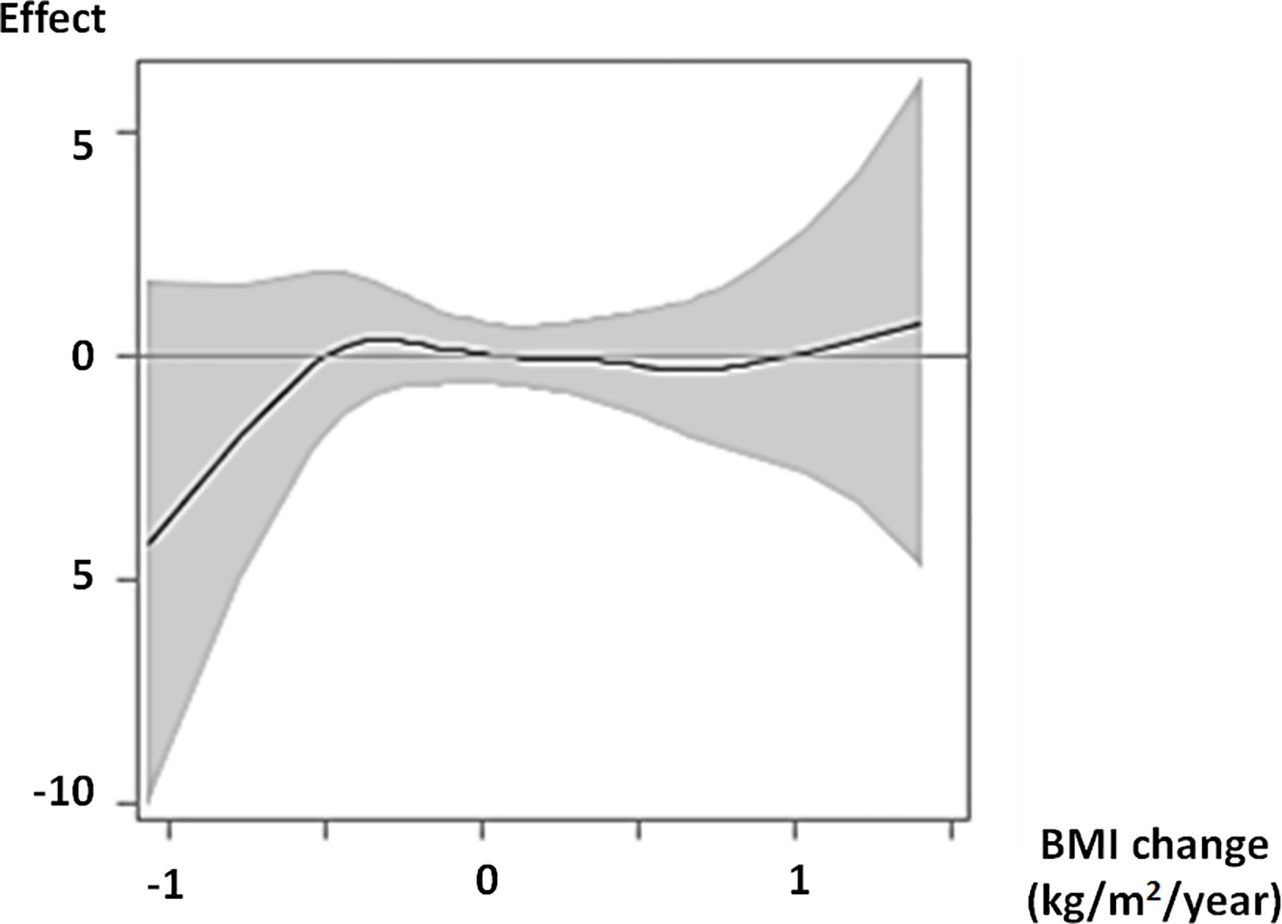
Effect of BMI change on eGFR change in females of Group 1. The curve from a generalized additive model with spline shows the pattern of the effect of BMI change on eGFR change with 95% confidence interval. Positive and negative values of the effect indicate increasing and decreasing eGFR changes, respectively. The models were adjusted for baseline characteristics. Abbreviations: Effect, effect of BMI change on eGFR change; BMI, body mass index; eGFR, estimated glomerular filtration rate.

In females, no statistically significant relationships between eGFR change, BMI, and BMI change were observed in all females (BMI, linear *p* = 0.61, spline *p* = 0.69; BMI change, linear *p* = 0.0045, spline *p* = 0.11) and any groups. In Group 1, eGFR change tended to decrease with a decreasing BMI change (linear *p* = 0.85, spline *p* = 0.11) (Fig 7).

## Discussion

In this study, among the healthy people, nonlinear relationships between eGFR change, BMI, and BMI change were investigated using scatter plots with spline and GAMs. In males, the relationships between BMI change and eGFR change varied with BMI. The overall trends of the associations between eGFR change, BMI and BMI change were u-shaped. The effects of BMI change on eGFR change in males with high normal BMIs (Group 3) were different from those in overweight males (Group 4). As far as we investigated, the different relationships between BMI changes and eGFR changes depending on BMI have never been reported. In females, although no statistically significant relationship was observed between BMI change and eGFR change, a rapidly decreasing eGFR change was observed with a decreasing BMI change in females with low BMIs. From these results, it is suggested that to evaluate the relationship between kidney function and BMI should be evaluated from the viewpoints of not only (1) BMI itself but also (2) BMI change.

Regarding the relationship between eGFR change and BMI, previous studies showed that obesity is associated with loss of kidney function and incident CKD [5–7]. On the other hand, the Reasons for Geographic and Racial Differences in Stroke (REGARDS) study showed that obese people (30 kg/m^2^ ≤ BMI) without metabolic syndrome have lower risks of end-stage renal disease (ESRD) than people with normal weight [15]. Metabolic syndrome is a confounder of the relationship between BMI and risk of ESRD. In this study, a u-shaped relationship between BMI and eGFR change was observed in males. eGFR tended to increase in males with low BMIs (< 22 kg/m^2^) and with high BMIs (28 kg/m^2^ ≤). The results of this study were in accordance with previous studies [5–7, 15]. That is, compared with medium BMIs, low and high BMIs may pose a lower risk of loss of kidney function. Because DM patients were not included in this study, similar results to the REGARDS study were observed. These results suggest that there may be some common metabolic factors between DM and metabolic syndrome that affect the loss of kidney function, such as insulin resistance, and hypertension, which affects kidney function [16, 17].

The benefits of weight loss for obese people have been reported. A weight-loss interventional study of ORG patients showed that the decreased-BMI group showed greater decrease in urinary protein level than the stable- and increased-BMI groups [18]. An animal study showed that calorie restriction prevents glomerular enlargement, podocyte hypertrophy, proteinuria, and glomerulosclerosis [19]. These studies suggest that weight loss may suppress histopathological changes of the kidney, which protects against impairment of the kidney function in obese people. It has been reported that in obese people, weight loss by surgical interventions improves kidney function and reduces blood pressure and microalbuminuria [20, 21]. Moderate weight loss by cointervention with diet and exercise is associated with improved eGFR [22]. A randomized controlled trial of obese people (27 kg/m^2^ < BMI) showed that creatinine clearance remains stable in the diet group and significantly worsens in the control group [23]. Our study showed that in overweight males (25 kg/m^2^ ≤ BMI), a decreasing BMI change was associated with an increasing eGFR change. These lines of evidence suggest that weight loss may be effective in improving and protecting kidney function in obese people.

Although the beneficial effects of weight loss on obese people has been observed, weight loss has been reported as a risk factor for incident CKD in males [11, 12]. A cohort study in Korea showed that the high risk of incident CKD is associated with weight loss among high-BMI males (23 kg/m^2^ ≤ BMI) [11]. Our study showed that eGFR tended to decrease with a decreasing BMI change in males with high normal BMIs (22 kg/m^2^ ≤ BMI < 25 kg/m^2^), which was in accordance with the study in Korea. Moreover, overweight males showed that their eGFR tended to increase with a decreasing BMI change, as described above. Our findings suggest that weight loss may improve eGFR in overweight males, but may aggravate eGFR in males with high normal BMIs. And the influence of BMI change on kidney function may be determined by a balance between benefits and harms of weight loss to eGFR, which is dependent on BMI.

The Korean study showed that in males with normal weight, a rapid weight loss (< -0.75 kg/year) is a risk factor for incident CKD [11]. In our study, males with high normal BMIs showed that a rapidly decreasing BMI change (< -1 kg/m^2^/year) was associated with a decreasing eGFR change, and that a slowly decreasing BMI (-1 to 0 kg/m^2^/year) was associated with an increasing eGFR change. On the other hand, for overweight males, instead of the speed of a decreasing BMI change, a decreasing BMI change was associated with an increasing eGFR change. These results suggest that the speed of weight loss may affect kidney function in males with high normal BMIs, and that slow weight loss is more beneficial for kidney function than rapid weight loss.

Serum creatinine level closely reflects skeletal muscle mass, and changes with it [24, 25]. Creatinine-level-based eGFR has a limitation, that is, the weight-loss-associated decrease in skeletal muscle increases eGFR apparently [26–28]. In this study, the males with low normal BMIs or who were underweight (BMI < 22 kg/m^2^) showed tendencies their eGFR tended to increase with a decreasing BMI change. From considering previous studies, these findings may reflect the decrease in muscle mass. On the other hand, in the males with high normal BMIs, this increasing eGFR change was not observed with a decreasing BMI change. A possible reason for this inconsistency was the actual decrease in kidney function caused by weight loss affected eGFR to a great extent than the apparent improvement of serum creatinine level due to the decrease in muscle mass. In the overweight males, instead of certain effects of decrease in muscle mass, the kidney function may have improved actually.

It has been reported that kidney function declines more rapidly in males than in females [29]. Sex hormones are an important factor for the loss of kidney function in males [30]. However, there are a few studies in which the relationship between BMI and loss of kidney function depending on gender was investigated. In males, high BMI or obesity has been reported as a risk factor of loss of kidney function [11, 31–33]. In females, the effect of obesity on the loss of kidney function has not been established yet. A longitudinal study showed that BMI is negatively associated with eGFR in males, but not in females [33]. On the other hand, the Coronary Artery Risk Development in Young Adults study showed that in both young males and females (average age, 35 years), high BMI is associated with a rapid decrease in eGFR [32]. Our study showed that BMI and BMI change were not associated with eGFR change in females. Although the results were not statistically significant, among underweight females, the decreasing BMI change was observed with a rapidly decreasing eGFR change. These findings suggest that BMI may be associated with kidney function in females, and that it would be more difficult to detect the effects of BMI on kidney function in females than in males. A prospective cohort study of high cardiovascular risk patients in Taiwan reported that neck circumference is associated with 24-h creatinine clearance rate and microalbuminuria [34]. Comprehensively evaluation using not only BMI but also anthropometric measurements such as neck circumference may be useful to evaluate the effects of weight changes on kidney function.

Our study had several limitations. First, the subjects of this study were healthy, and those with missing values were excluded from the study, which might have resulted in a selection bias. Second, the subjects who were not followed up were excluded from the analysis targets of the longitudinal study. Moreover, the accurate date of onset of loss of kidney function was unclear. Therefore, the risk of the progression of the loss of kidney function might have been underestimated. Third, the analysis dataset contained data based on two points, baseline and 3 years later. We were unable to evaluate the relationship between eGFR change and BMI change for a long time using repeatedly measured data. Fourth, because this was an observational study, the preventive effect of BMI change on kidney function has remained unclarified. Fifth, as discussed above, GFR was estimated from serum creatinine level, which is affected by muscle mass. Sixth, the scatter plots suggest that the relationships between eGFR and BMI change in old males differed from those in young males. However, no interaction between age and BMI change was observed in males. The reasons for these findings may be as follows: (1) the sample size of males was not sufficiently large, and (2) the mean age of males was 40.11±9.49 years, and elderly persons were not included in this study. The range of ages in this study may be too small to show the effects of age on the relationship between eGFR change and BMI change.

## Conclusions

In this study, rapid weight loss was associated with the loss of kidney function in males with normal weight, and with improvement of kidney function in overweight males. The risks and benefits of weight loss in relation to kidney function may differ depending on BMI and weight loss speed in males. Therefore, the effects of BMI and BMI change on kidney function should be taken into consideration when controlling weight.
